# Improving “color rendering” of LED lighting for the growth of lettuce

**DOI:** 10.1038/srep45944

**Published:** 2017-04-03

**Authors:** Tao Han, Vitaliy Vaganov, Shixiu Cao, Qiang Li, Lili Ling, Xiaoyao Cheng, Lingling Peng, Congzhi Zhang, Alexey N. Yakovlev, Yang Zhong, Mingjing Tu

**Affiliations:** 1 Chongqing Engineering Research Center for Optoelectronic Materials and Devices, Research Institute for New Materials Technology, Chongqing University of Arts and Sciences, Chongqing 402160, People’s Republic of China; 2 Institute of High Technology Physics, Tomsk Polytechnic University, Tomsk 634050, Russia; 3 Citrus Research Institute, Southwest University, Chongqing 400712, People’s Republic of China

## Abstract

Light plays a vital role on the growth and development of plant. On the base of white light with high color rendering to the benefit of human survival and life, we proposed to improve “color rendering” of LED lighting for accelerating the growth of lettuce. Seven spectral LED lights were adopted to irradiate the lettuces under 150 μmol·m^−2^·s^−1^ for a 16 hd^−1^ photoperiod. The leaf area and number profiles, plant biomass, and photosynthetic rate under the as-prepared LED light treatments were investigated. We let the absorption spectrum of fresh leaf be the emission spectrum of ideal light and then evaluate the “color rendering” of as-prepared LED lights by the Pearson product-moment correlation coefficient and CIE chromaticity coordinates. Under the irradiation of red-yellow-blue light with high correlation coefficient of 0.587, the dry weights and leaf growth rate are 2–3 times as high as the sharp red-blue light. The optimized LED light for lettuce growth can be presumed to be limited to the angle (about 75°) between the vectors passed through the ideal light in the CIE chromaticity coordinates. These findings open up a new idea to assess and find the optimized LED light for plant growth.

Light is the primary environmental factor for plant growth and development. Caused by lack of sunshine, plant growth in northern countries and during non-native seasons has been already supported by artificial illumination^1^,^2^. Artificial light sources such as incandescent, metal-halide, fluorescent lamps are generally used for plant cultivation. But some disadvantages, including high energy consumption, short lifetime, and uncontrollable wavelengths out of photosynthesis, have influenced their application. Recently, Light-emitting diodes (LEDs), which are regarded as the new-generation lighting source, have been applied widely owing to their high brightness, long lifetime, environmental friendliness and spectral manipulation^3^. Spectral modulation produced by LED lighting plays an important role for enhancing plant development, because plant morphology and physiology are strongly influenced by the light quality, which refers to the color or wavelength reaching a plant’s surface^4^.

In many reports^5^,^6^,^7^,^8^, red–blue LEDs were demonstrated to be more suitable for lettuce cultivation. Red and blue lights have the greatest impact on plant growth because they are the major energy sources for photosynthetic CO_2_ assimilation. But Kim *et al*. found the addition of green light to red-blue LEDs enhanced lettuce growth and produced more biomass^9^. The effects of light quality are more complex, and mixed results were often reported. Additionally, there are inconstant spectral demands and photosynthetic responses for different plant species. However, spectral distributions are not yet custom-made and optimized. An optimal strategy of light quality regulation would allow one to select the most efficacious spectral composition, thereby enabling plant growth in the most energy-efficient way.

It is well known the white LEDs with high color rendering are conductive to human eyes. In this paper, we borrowed the concept of “color rendering” and tried to enhance the “color rendering” of LED lighting for the growth of plant subject to the absorption spectrum of lettuce. The leaf area and number profiles, plant biomass, and Photosynthetic rate (Pn) under the as-prepared LED light treatments were examined. We suppose the absorption spectrum of fresh leaf to be the emission spectrum of ideal light^10^, and then evaluate the “color rendering” of as-prepared LED lights by the Pearson product-moment correlation coefficient and CIE chromaticity coordinates.

## Results

### LED light strategy

**Figure 1:**
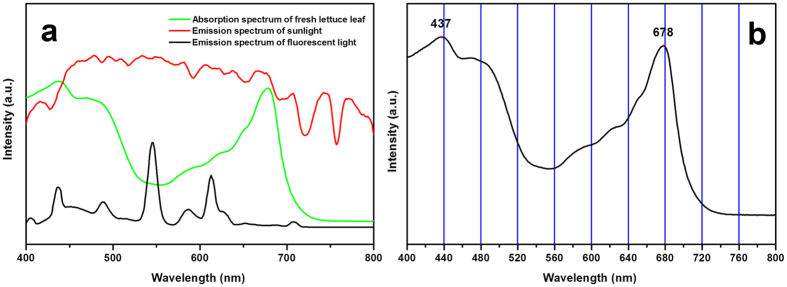
Comparison of absorption spectrum of fresh lettuce leaf with spectra of sunlight and white fluorescent light (**a**) and absorption spectrum of the fresh leaf (**b**).

Until today fluorescent lamps are used as supplementary lighting for plant growth. However, fluorescent light does not fit so well with the absorption of plants. Plant pigments have specific wavelength absorption patterns known as absorption spectra. Figure 1 shows the comparison of absorption spectrum of fresh lettuce leaf with spectra of sunlight and white fluorescent light. There are two intense absorption bands of fresh lettuce leaf in the range of 400–800 nm, mainly from chlorophyll and carotene absorptions^11^. The 437 nm bands belong to blue light for developing the chloroplast and the 678 nm bands are attributable to red light for promoting photosynthesis^12^,^13^,^14^,^15^. But a valley centered at ~550 nm indicates that yellow light probably has a negative effect on the growth of plants^16^,^17^. Sunlight covering the visible region shows the best color rendering for human eyes, and yet the yellow light may slow down the plant growth^16^,^17^. Unlike sunlight, the spectrum of fluorescent light as a typical artificial light is discrete, lack of red component and excessive yellow light. Nonetheless, in the green-yellow light region there are several weak absorption bands, which could act synergistically.

**Figure 2:**
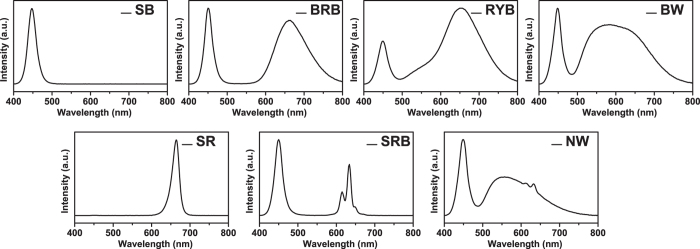
Spectra of the as-prepared LED lights.

Therefore, we assume the ideal light for the growth of lettuce should have an emission spectrum to overlap or contain in the absorption spectrum of lettuce. In light of color rendering for human eyes, we define the suitable light for the growth of plant as light with good “color rendering”. To verify our hypothesis, we designed and did a proof-of-concept experiment. The LED lights with various colors were fabricated by combining blue chip and complex phosphors. Figure 2 shows spectra with different shape of the as-prepared LED lights. The spectra include sharp blue (SB), broad red-blue (BRB), red-yellow-blue (RYB), broad white (BW), sharp red (SR), sharp red-blue (SRB), and narrow white (NW) light. Herein, SRB light is used to mimic the light of red–blue LED, as shown in Figure S1. In comparison, RYB light is distinctly similar to the ideal spectrum, which may be the light with good “color rendering” for the growth of plant.

### Growth and morphology

**Figure 3:**
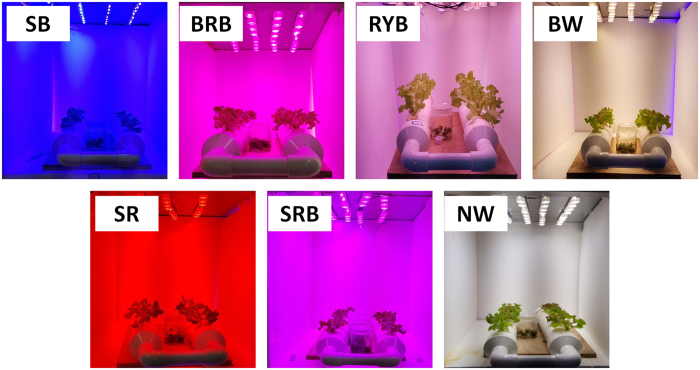
Chambers installed with the as-prepared LED lights.

**Figure 4:**
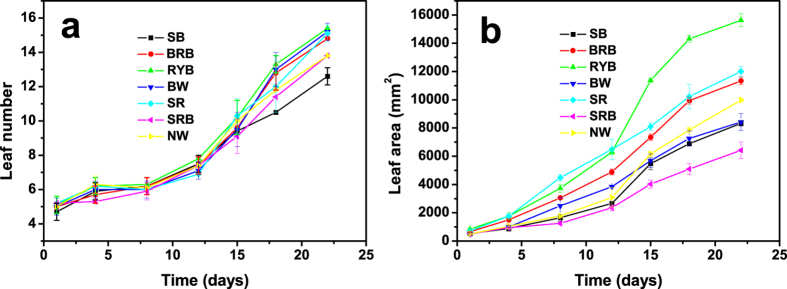
Growth rates of leaf number (**a**) and leaf area (**b**) under the as-prepared LED lights. Data are mean values (n = 10) ±SE. Error bars represent the SE.

**Figure 5:**
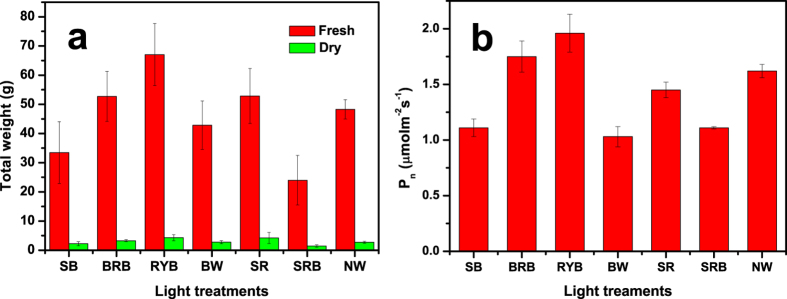
Dependence of fresh and dry weight (**a**) and P_n_ (**b**) on the as-prepared LED lights treatments (on the 22^th^ days). Data are mean values (n = 5) ±SE. Error bars represent the SE.

Figure 3 shows at day 8 the growth images of lettuce plants under the as-prepared LED light treaments. It can be seen that all the plants are alive and well, and the plants grow obviously bigger under RYB light treament than others. To further reveal the growth of lettuce, the growth rates of the leaf number and leaf area under the as-prepared LED light treatments in 22 days were shown in Fig. 4. All the profiles have “S” shape profiles and the plants accelerate to grow from the 12^th^ day, whereas the leaf number grows faster under the red-yellow-blue light, and leaf areas expand more rapidly under the treaments of RYB light, SR light and BRB light than others. Red light induces hypocotyl elongation and cotyledon expansion in seedlings, but blue light suppresses hypocotyl elongation^18^. Hence, the sharp red light condition has higher growth rate and lager leaf area than the samples grown under blue and red light and some similar results had been reported^4^,^19^. Especially, the leaf area of lettuce under the radiation of RYB light is 2.4 times as high as that under the radiation of SRB light. In addition, the similar trends of the fresh and dry weight and P_n_ under the as-prepared LED light treatments were shown in Fig. 5. The dry weight of lettuces under the RYB light treament is 3.0 times as high as that under the radiation of SRB light. The results can support the above deduction that the RYB light with good “color rendering” is to the benefit of the growth of plant.

## Discussion

**Figure 6:**
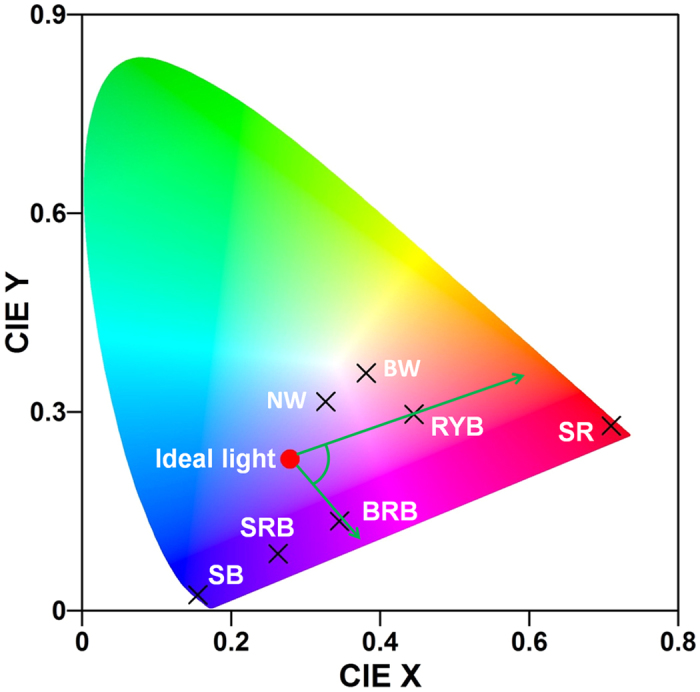
CIE chromaticity coordinates of ideal light for lettuce growth and the as-prepared LED lights.

To get the quantitative data for evaluating spectral similarity between the as-prepared LED lights and the ideal light^20^,^21^, we introduced the Pearson product-moment correlation coefficient (a value between −1 and +1) that characterize how strongly two variables are related to each other. If the value of *r* is close to +1, this indicates a strong positive correlation, and if *r* is close to -1, this indicates a strong negative correlation. The Pearson product-moment correlation coefficient (*r*) for two sets of values, *x* and *y*, is given by formula (1),


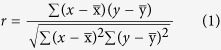


where *x* is the spectral data points of each as-prepared LED light and *y* is those of the ideal light. We divided the absorption spectra of the lettuce leaf (Fig. 1b) into ten intervals and calculated correlation coefficients, respectively. Considering the fact, there is no effect for plant growth without light, and no obvious negative correlation with different emission intensity in the same interval, so the correlation coefficients were given to zero in these cases. All the spectral data points were plugged into the formula (1) and then the correlation coefficients were obtained. Table 1 shows the correlation coefficients of the as-prepared LED lights related to the ideal light for lettuce. The results show the RYB light has the high correlation coefficient of 0.587, whereas it is lower than that of BRB light. It can be explained that the negative correlation increases by adding the yellow light in BRB light. The other results do not agree well with their correlation coefficients, due to more efficient photosynthesis under the red light than others. In another way, the CIE chromaticity coordinates were selected to assess and predict the optimized LED light for plant growth. Figure 6 shows the CIE chromaticity coordinates of ideal light for lettuce growth and the as-prepared LED lights. The distances between the as-prepared LED lights and the ideal light in CIE chromaticity coordinates were calculated in Table 2. It can be easy to explain that the lettuces grow faster under BRB light than SRB light, but under NW light than BW light, because the distances are closer to the ideal light in the CIE chromaticity coordinates. The electrical power consumptions of as-prepared LED beads were estimated by the photoelectric conversion efficiency, as shown in Table 2. The photoelectric conversion efficiency of LED declines due to combining the various phosphors. Summarizing the above results, all the lights absorbed by plant are not useful to enhance the growth, so the ideal spectrum is not the optimized spectrum for plant growth. Using the CIE chromaticity coordinates, we can presume the optimized LED light for lettuce growth will be confined to the angle (about 75°) between the vector from ideal light to RYB light and the vector from ideal light to BRB light.

By employing the Pearson product-moment correlation coefficient and CIE chromaticity coordinates, in this report we open a new avenue to estimate the LED lights for plant growth. The results show under the radiation of RYB light the leaf area growth rates and dry weight of lettuce is 2.4 and 3.0 times as high as that under the radiation of SRB light, respectively. The lettuces grow faster under BRB light than SRB light, but faster under NW light than BW light, because the distances are closer to the ideal light in the CIE chromaticity coordinates. These findings show a promising way to presume the optimized LED light for plant growth.

## Methods

### Plant materials and experimental setup

Lettuce seeds were sown in pearlite and geminated under 150 μmol·m^−2^·s^−1^ irradiation of white LED. Uniform-sized seedlings of lettuce at the 5-leaf stage were randomly divided into seven groups and individually transplanted in a polyvinyl chloride tube with ten holes. The polyvinyl chloride tubes were placed in a growth chamber. The chambers with the size of 550 mm in length, 450 mm in width, and 450 mm in height were installed with seven LED lights with different colors and spectral components, as shown in Figs 2 and 3.

The polyvinyl chloride tubes were pumped in aerated complete nutrient solution adjusted to pH6 and an electrical conductivity of 1.1 mScm^−1^. The nutrient solution was placed in a big closed container and circulated every day for renewing that in the polyvinyl chloride tubes. The photoperiod was maintained at 16 h. The plants were irradiated with the LED light treatments and harvested at 22 days after transplanting. The air temperature, relative humidity, photosynthetic photon flux density and CO_2_ levels for all treatments were respectively maintained throughout the experiment at 24 °C (day and night), 75%, 150 μmol·m^−2^·s^−1^ and 380–400 ppm in the growth chambers.

### Growth and morphology measurement

Morphological characteristics such as leaf number, leaf area, fresh height, and dry weight were tested. The leaf area (cm) of every plant was measured on fresh leaves by paper weighing method^22^,^23^. Growth rates of leaf area and leaf number were determined during the experimental period (22 days). The samples were dried in a drying oven for 48 h at 70 °C before weighing. P_n_ was performed on a single leaf exposed to as-prepared LED lights provided by a portable photosynthesis system (Li-6400, Li-CorInc., Lincoln, NE, USA).

### LEDs fabrication and spectral characterization

The LEDs with various spectra were fabricated by combining blue chip (450 nm, 1 W) and CaAlSiN_3_:Eu^2+^ red phosphors, K_2_TiF_6_:Mn^4+^ red phosphors or Y_3_Al_5_O_12_:Ce^3+^ yellow phosphors^24^,^25^. The blue chip was first fixed on the bottom of a reflector. The phosphors were mixed with silicone thoroughly and the obtained phosphor–silicone mixture was coated on the surface of the LED chips. The LEDs were assembled on an Aluminum substrate and added concave lens to produce different lights. Of these, SB and SR lights were generated by blue LEDs and red LEDs, respectively, but other lights were acquired by phosphor-conversion LEDs. The photoelectric properties of the fabricated devices were measured by an spectroradiometer system (HSP3000, Hongpu, China). The absorption spectrum of fresh lettuce leaf was aquired by recording the diffusion reflection taken on an UV-vis spectrometer (Agilent 8453, Agilent, USA). Sunlight and fluorescent light, and photosynthetic photon flux density were analyzed by an spectroradiometer system (PLA-20, Everfine, China).

## Additional Information

**How to cite this article:** Han, T. *et al*. Improving “color rendering” of LED lighting for the growth of lettuce. *Sci. Rep.* **7**, 45944; doi: 10.1038/srep45944 (2017).

**Publisher's note:** Springer Nature remains neutral with regard to jurisdictional claims in published maps and institutional affiliations.

## Supplementary Material

Supplementary Figure S1

## Figures and Tables

**Table 1:** Correlation coefficients of the as-prepared LED lights related to the ideal light for lettuce.

Spectral interval	LED light						
	SB	BRB	RYB	BW	SR	SRB	NW
400–440 nm	0.859	0.844	0.919	0.914	0.000	0.858	0.903
441–480 nm	0.770	0.542	0.663	0.736	0.000	0.645	0.700
481–520 nm	0.794	0.801	0.000	0.000	0.000	0.826	0.000
521–560 nm	0.000	0.000	0.000	0.000	0.000	0.000	0.000
561–600 nm	0.000	0.888	0.949	0.472	0.000	0.842	0.000
601–640 nm	0.000	0.986	0.990	0.000	0.874	0.756	0.000
641–680 nm	0.000	0.190	0.000	0.000	0.146	0.000	0.000
681–720 nm	0.000	1.000	0.961	0.999	0.899	0.864	0.996
721–760 nm	0.000	0.935	0.935	0.938	0.000	0.000	0.934
761–800 nm	0.000	0.464	0.457	0.424	0.000	0.000	0.490
Average value	0.242	0.665	0.587	0.448	0.192	0.479	0.402

**Table 2:** Distances between the as-prepared LED lights and the ideal light in CIE chromaticity coordinates and photoelectric conversion efficiency.

LED light	CIE (x, y)	Distances in chromaticity coordinates	Photoelectric conversion efficiency
SB	0.1548, 0.0239	0.240	0.470
BRB	0.3446, 0.1353	0.115	0.299
RYB	0.4448, 0.2967	0.179	0.273
BW	0.3814, 0.3594	0.165	0.336
SR	0.7096, 0.2787	0.433	0.399
SRB	0.2631, 0.0861	0.144	0.325
NW	0.3270, 0.3160	0.0989	0.386
