# Supplementary material for: Improving “color rendering” of LED lighting for the growth of lettuce
Source: Sci Rep. 2017 Apr 3;7:45944. doi: 10.1038/srep45944 (PMC5377472; doi:10.1038/srep45944)
Supplement: Supplementary Figure S1 [file srep45944-s1.pdf]

Improving "color rendering" of LED lighting for the growth of lettuce

Tao Han[1], Vitaliy Vaganov[3], Shixiu Cao[1], Qiang Li[1], Lili Ling[2], Xiaoyao Cheng[1], Lingling Peng[1], Congzhi Zhang[1], Alexey N. Yakovlev[3], Yang Zhong[3], Mingjing Tu[1]

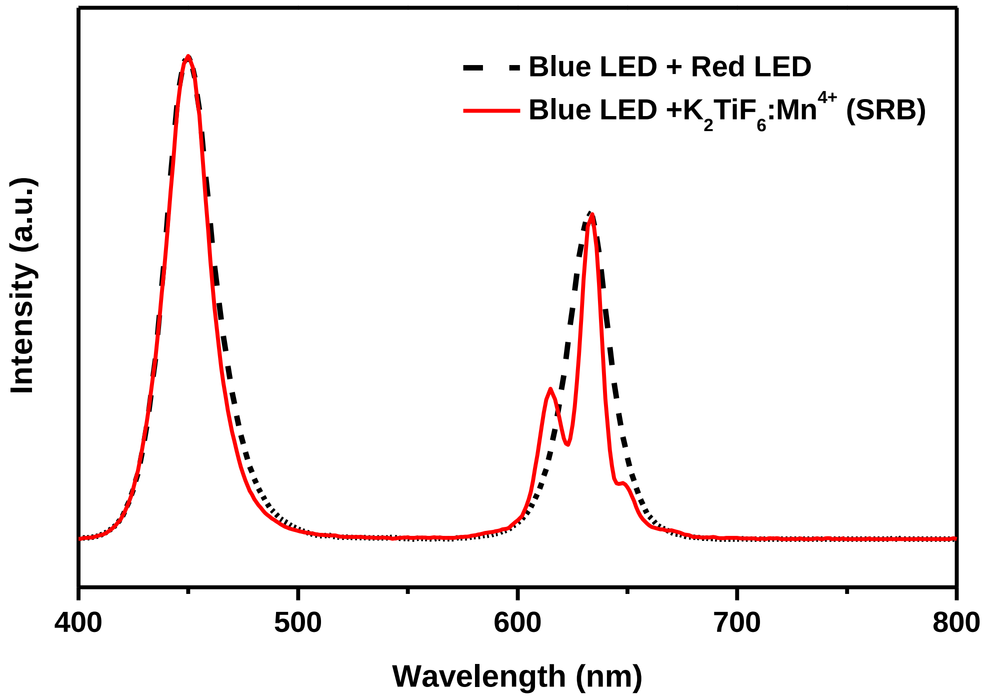

Figure S1 Spectral comparison of red–blue LED and SRB lights
